# Concise Review: Mesenchymal Stromal Cell‐Based Approaches for the Treatment of Acute Respiratory Distress and Sepsis Syndromes

**DOI:** 10.1002/sctm.16-0415

**Published:** 2017-01-09

**Authors:** Christian L. Johnson, Yorick Soeder, Marc H. Dahlke

**Affiliations:** ^1^ Department of SurgeryUniversity Hospital RegensburgRegensburgGermany

**Keywords:** Mesenchymal stromal cells, Sepsis syndrome, Acute respiratory distress syndrome, Immunomodulation, Cell therapy

## Abstract

Despite extensive research on candidate pharmacological treatments and a significant and increasing prevalence, sepsis syndrome, and acute respiratory distress syndrome (ARDS) remain areas of unmet clinical need. Preclinical studies examining mesenchymal stromal cell (MSCs) based‐therapies have provided compelling evidence of potential benefit; however, the precise mechanism by which MSCs exert a therapeutic influence, and whether MSC application is efficacious in humans, remains unknown. Detailed evaluation of the limited number of human trials so far completed is further hampered as a result of variations in trial design and biomarker selection. This review provides a concise summary of current preclinical and clinical knowledge of MSCs as a cell therapy for sepsis syndrome and ARDS. The challenges of modeling such heterogeneous and rapidly progressive disease states are considered and we discuss how lessons from previous studies of pharmacological treatments for sepsis syndrome and ARDS might be used to inform and refine the design of the next generation of MSC clinical trials. Stem Cells Translational Medicine *2017;6:1141–1151*

**Significance Statement**

Mesenchymal stromal cells (MSCs) are increasingly being assessed as therapeutic for a range of immunological diseases. The present report provides an analysis of the current preclinical and clinical knowledge of MSCs as a potential cell‐based treatment for sepsis syndrome and acute respiratory distress syndrome.

## Introduction

Sepsis is a potentially lethal syndrome that can develop following an infection in which a breakdown in immune homeostasis results in both pro‐inflammatory and anti‐inflammatory mechanisms becoming uncoupled from normal regulation 1. Despite decades of research, sepsis syndrome remains a global health concern with no causal treatment. Current estimates indicate that there are in excess of 19 million new cases worldwide per year 2. While the prevalence of severe sepsis (sepsis accompanied by acute organ dysfunction) on ICU's of western countries is comparable (USA 11.8% 3, Germany 11.0% 4, Italy 11.6% 5), it is likely disproportionally higher in low‐ and middle‐income countries 6. Sepsis syndrome ranks as the leading cause of death in hospitalized patients 7 with mortality rates ranging from between 20 and 50% 4, 8. Sepsis is inherently heterogeneous and its treatment represents a significant challenge. Clinical outcomes are influenced by individual patient factors, including age, gender and race 8 and by both the infection agent and the site of clinical manifestation. Currently there is no gold‐standard diagnostic test to predict severity and to guide patient treatment 9. Although sepsis syndrome is normally considered to be the clinical response to a suspected or proven infection 10, it can also occur following sterile tissue injury 7. Despite a recent proposal 9 to revise the previous consensus definition of sepsis and septic shock 11 that recommended removal of the term “severe sepsis” and replacement of SIRS criteria in sepsis diagnosis with the “Sequential Organ Failure Assessment” (SOFA) and quick SOFA (qSOFA) (Table 1) score systems, concerns have been raised that there remains a disproportionate emphasis on infection 12, 13.

**Table 1:** The changing clinical definitions of sepsis

ACCP/SCCM Consensus definitions (1992) [89]	International consensus definitions (2001)[11]	Third international consensus definitions (2016)[9]
**Systemic inflammatory response syndrome (SIRS)**: Manifestation of two or more of the following: Temperature >38°C or <36°C Heart rate >90 bpm Respiratory rate >20/min or PaCO2 <32 mmHg White blood cell count >12,000/cu mm, <4,000/cu, or >10% immature bands. **Sepsis**: SIRS + suspected or documented infection. **Severe sepsis**: Sepsis associated with organ dysfunction, hypoperfusion, or hypotension. Hypoperfusion andperfusion abnormalities may include, but are not limited to: Temperature >38°C or <36°C Lactic acidosis Oliguria Alteration of mental status **Septic shock**: Sepsis induced withhypotension despite adequate fluidresuscitation.	Clinical evidence of infection and some of the following: General variables Fever or hypothermia Heart rate >90 beats per minute Elevated respiratory rate Alteration of mental status Significant edema or positive fluid balance Hyperglycemia in the absence of diabetes Inflammatory variables Leukocytosis, leukopenia, or normal WBC count with >10% immature forms Elevated plasma C‐reactive protein Procalcitonin Hemodynamic variables Arterial hypotension Mixed venous oxygen saturation >70% Elevated cardiac index Organ dysfunction variables Arterial hypoxemia Acute oliguria or creatinine increase Coagulation abnormalities Ileus Thrombocytopenia Hyperbilirubinemia Tissue perfusion variables Hyperlactatemia Decreased capillary refill or mottling	**SIRS**: No longer used. **Sepsis**: life‐threatening organ dysfunction caused by a dysregulated host response to infection. Fulfilling both qSOFA: Respiratory rate ≥22/min Altered mentation Systolic blood pressure ≤100 mmHg with an acute increase of ≥2 SOFA points. **Severe sepsis**: No longer used. **Septic shock**: A subset of sepsis in which underlying circulatory and cellular/metabolic abnormalities are profound enough to substantially increase mortality. Vasopressor therapy needed to elevate MAP ≥65 mmHg and lactate >2 mmol/l (18 mg/dl) despite adequate fluid resuscitation.

## Pathogenesis

In sepsis syndrome, an inappropriate immune response can persist after resolution of the initial causal infection. This is primarily driven by the innate immune response to intracellular, extracellular or pathogen‐associated danger signals termed “Damage‐associated molecular pattern molecules” (DAMPs), for example high mobility group box 1 (HMGB1) protein, or “Pathogen‐associated molecular pattern molecules” (PAMPs). DAMPs and PAMPs interact with pattern‐recognition receptors including toll‐like receptor (TLRs), C‐type lectin receptors, NOD‐like receptors (NLRs) and RIG‐I‐like receptors (RLRs). Activation of NLRs and RLRs promote the assembly of inflammasomes 14 which mediate the release of inflammatory cytokines including TNF‐α, IL‐1, IL‐6, IL‐18, and HMGB1, which itself also possesses an inflammatory cytokine function in sepsis 15. Normally anti‐inflammatory mediators act to dampen the potentially deleterious inflammatory cascade. The neuroinflammatory reflex stimulates secretion of acetylcholine by CD4^+^ T cells and norepinephrine release within the spleen leading to the inhibition of macrophage‐derived pro‐inflammatory cytokines. These vital feedback mechanisms appear impaired during the onset of sepsis. It has been shown that patients who survive early sepsis but subsequently remain dependent on intensive care have evidence of increased immunosuppression 16. Patients with severe sepsis can also present a diminished response to PAMPs and DAMPs 17. Organ failure in severe sepsis is speculated to result from disruption of epithelial and endothelial barriers, indeed HMGB1 has been shown to enhance epithelial permeability. This hypothesis is supported by the observation that organ dysfunction in severe sepsis is characterized by cellular substrates in the serum 7. Notably, the primary host responses to infection appear similar to that of sterile inflammation and ischemic reperfusion injury 18, providing a possible explanation for sepsis of a noninfectious origin or mixed forms of sepsis syndrome. Sepsis can promote the onset of a variety of organ specific complications including acute kidney injury (AKI), acute liver injury, myocardial dysfunction, acute lung injury (ALI), and acute respiratory distress syndrome (ARDS). The study of ALI and ARDS, in which unregulated inflammation focused in the lungs leads to a breakdown of the pulmonary capillary endothelial barriers and results in fluid accumulation, will be considered in further detail within this review.

## Current Treatment Regimens

Treatment of sepsis syndrome places a significant burden on healthcare infrastructure. In the USA alone, annual primary treatment costs in 2007 were estimated at $24.3 billion 19. Current evidence‐based treatment recommendations are published by the Surviving Sepsis Campaign 20 and consist primarily of a resuscitation bundle (to be completed within 6 hours) and a management bundle (to be completed within 24 hours). The resuscitation bundle is composed of; (a) a rapid source control and administration of empirical broad spectrum antibiotics and if necessary vasopressors and fluid resuscitation, (b) an early goal‐directed therapy (EGDT) to achieve target values for central venous pressure, mean arterial pressure, urine output, both central venous and arterial oxygen saturation, hematocrit, cardiac index, and systemic oxygen consumption. The continued application of current treatment guidelines has led to improved algorithms for treating septic patients; however alone these may be insufficient to produce further decreases in sepsis mortality rates. For example, while antibiotic treatment is a key pharmacological intervention in sepsis management, the rising incidence of antibiotic resistant microbes is recognized as an emerging obstacle 10. Furthermore, a recent meta‐analysis 21 on the impact of EGDT has called into question the benefits of this therapeutic approach. The authors reported that EGDT was not superior to standard of care for septic shock patients but was associated with both an increased admission to ICU and an increased utilization of ICU resources. Despite clinical trials examining potentially causal therapeutic compounds for sepsis treatment, including an IL‐1 receptor antagonist 22, TNF‐α antagonist 23, human recombinant activated protein C (APC) 24, intravenous immunoglobulin G therapy 25, TLR4 antagonist 26, and talactoferrin 27, no new pharmacological therapies have entered the clinical routine 28.

## Preclinical Sepsis Models

Small animal models have been used extensively to investigate the physiological process that lead to sepsis syndrome and ALI/ARDS, and to study the effects of potential therapeutics. Generally, these models introduce a systemic or a localized challenge into the host in order to induce a sepsis‐like pathology 29. In systemic challenge models, bacteria (i.e., *Escherichia coli*) or bacterial‐derived toxins (i.e., lipopolysaccharide, LPS) are administered into the animal by intravenous or intraperitoneal injection. The resulting rapid systemic release of pro‐inflammatory cytokines, such as TNF‐α and IL‐1, and an increase in hypodynamic cardiovascular activity, leads to onset of a septic shock‐like state. In localized challenge models, a source of infection is introduced into a specific anatomical region. As the lung and abdominal cavity are the most commonly observed locations of infection in septic patients 30, models of pneumonia and peritonitis are frequently used. Endotoxemia models utilizing LPS have been used extensively as they offer a convenient, reproducible method of experimentally inducing sepsis. However, the severity of LPS‐induced sepsis can depend on the model species or strain being used. For example, a much higher equivalent LPS dose is required to induce a sepsis‐like condition in mice than in human 31; therefore, such models are unlikely to accurately reflect the human disease course. The cecal ligation and puncture (CLP) model is chiefly used in the study of abdominal sepsis as it is thought to closely mimic the clinical situation 32, comprising of both a tissue trauma and a mixed microbial infection. In CLP, the cecum is ligated distal to the ileocecal valve; puncturing of the ligated cecum then permits contamination of the peritoneal cavity with colonic‐derived bacteria leading to the onset of an abdominal sepsis‐like condition. An added advantage of CLP is that it is highly adaptable, both sepsis onset and severity can be manipulated by varying the frequency of cecum punctures, needle size and the length of the cecum ligated 29.

## Mesenchymal Stromal Cells

Mesenchymal stromal cell (MSC) are non‐hematopoietic, multipotent stromal precursor cells that can be isolated from tissues such as bone marrow, adipose, dental pulp, placenta, cord blood, and matrix 33. MSC are capable of modulating the immune response 34 by both cell‐to‐cell contact and through the release of soluble paracrine factors including nitric oxide, indoleamine 2,3‐dioxygenase, PGE_2_, TGF‐β, and IL‐10 35, 36. MSC also promote expansion of the regulatory T cell (Treg) compartment 37. In a model of solid organ transplantation, we 38 have identified that infusions of MSC and multipotent adult progenitor cell (MAPCs), a bone marrow derived cell that shares a number of MSC characteristics including multipotency and immunosuppressive potential, lead to an induction of myeloid‐derived suppressor cells (MDSCs) to initially promote induction of pro‐inflammatory Th17 followed by conversion into CD4^+^ Tregs. The last decade has witnessed a surge in the number of preclinical and early phase clinical trials studying multipotent cell‐based technologies for indications including, but not limited to, GvHD, ischemic stroke, Crohn's disease, motor neuron disease, and acute myocardial infarction. MSC possess a number of characteristics that make them an attractive therapeutic candidate; they can undergo extended expansion without detriment to their multipotency or self‐renewal properties 39 and they exhibit low immunogenicity 40 and low tumorigenicity 41. Our own mouse models have provided evidence that intravenously infused MSC are short‐lived, with the lungs acting as the principle site of early entrapment 42. This may be a benefit in those indications in which the lung is the principle region of tissue injury, such as in the case of ALI/ARDS, as this would facilitate a high local concentration of MSC directly at the site of inflammation. Furthermore, a rodent model of ALI has provided initial evidence that the route of cellular application (intravenous and intratracheal) can partially influence MSC activity 43. However, it also might be counterproductive to apply MSC to patients where the pulmonary circuit is partially compromised as the potential for pulmonary embolism might be greater. MSCs are generally considered amendable to cryogenic storage 44. Notwithstanding, there is evidence that senescence is enriched in MSC populations that have been subjected to freeze‐thawing protocols 45 which may subsequently impair their immunosuppressive potency 46. To date, the majority of pilot and early phase clinical studies have focused on autologous or syngeneic MSCs. However, the process of isolating, cultivating, and assessing patient‐specific MSCs typically requires weeks, therefore prohibiting their use in those diseases with restrictive treatment windows. Consequently, studies are beginning to focus on allogeneic MSC as these cells would permit on‐demand patient treatment. Currently, our own group is conducting a phase I clinical trial to assess the safety and feasibility of third‐party MAPCs in liver transplant recipients 47. Recently, positive results have been reported in a phase III randomized, double blind, multicenter trial designed to assess the safety and efficacy of a proprietary allogenic adipose‐derived MSC (ADSC) to treat complex perianal fistulas in Crohn's disease (ClinicalTrials.gov NCT01541579). Here, the authors reported that a significantly greater proportion of ADSC treated patients achieved the primary endpoint of combined remission at week 24 compared to the placebo group 48. Certainly, the use of third‐party “off‐the‐shelf” MSC‐based would potentially enable an early therapeutic intervention such as is currently recommended in the treatment of sepsis syndrome.

## MSCs Reduce Inflammation

In vivo models of sepsis and ALI/ARDS have shown MSCs treatment to improve survival and to positively influence a number of indicators of the clinical course (Table 2). However, the precise mechanisms by which MSCs may mediate their effects remain for the most part unclear. Several preclinical studies have determined that in sepsis and ALI/ARDS, MSC exposure resulted in a decline in pro‐inflammatory cytokines including IL‐1α, IL‐1β, IL‐6, IFN‐γ, and TNF‐α 46, 49, 50, 51, 52, 53, 54, 55, 56, 57, 59, 61, 63, 64, 65, 66, 67, 68, 69, 70, 73 and an increase in anti‐inflammatory cytokines such as IL‐4, IL‐5, IL‐10 49, 50, 51, 56, 57, 63, 64, 67, 70, 73. Gupta et al. 49 found that in mice, intrapulmonary administration of BMSC 4 hours after induction of ALI by *E. coli* endotoxin resulted in improved survival, reduced excess lung water and improved lung histology, resulting in a decrease of bronchoalveolar lavage (BAL) TNF‐α and MIP‐2 and an increase of IL‐10 levels within BAL and plasma samples. Importantly in the context of ALI/ARDS, BMSC treatment resulted in a decrease in alveolar epithelium permeability. Animals treated with fibroblasts or apoptotic MSCs showed no such shift. It was also determined that MSC engraftment occurred infrequently and that although MSCs do express the LPS receptor complex, its presence did not alter endotoxin distribution. As invitro transwell‐experiments demonstrated that MSC were able to inhibit TNF‐α production by alveolar macrophages through a contact‐independent mechanism, the authors concluded that the beneficial effects of MSC in this model appeared to be largely mediated by soluble factors. In a CLP model of sepsis, Nemeth et al. 51 reported that an injection of 1 × 10^6^ BMSC resulted in increased survival, decreased vascular permeability, a reduction of TNF‐α and IL‐6 and an increase in IL‐10. No such effect was seen following injection of fibroblasts, whole bone marrow or heat‐killed BMSC. While a beneficial response to MSC could be observed in mice lacking mature T and B cells or NK cells, clodronate depletion of monocytes and macrophages abolished the MSC effect. Similarly, the MSC effect was abrogated in mice treated with either anti‐IL‐10 or anti‐IL‐10 receptor antibodies. The authors demonstrated that LPS treatment of BMSC resulted in an upregulation of PGE_2_ which in turn stimulated macrophage production of IL‐10. Li et al. 61 showed that after induction of ALI by LPS in rat, human umbilical cord derived MSC (UC‐MSC) improved survival, lung histology, wet‐dry weight ratio and reduced neutrophil infiltration. However, while UC‐MSC led to a reduction in serum contractions of TNF‐α, IL‐1β, and IL‐6 following LPS challenge, no effect was observed on IL‐10 levels. Sepúlveda et al. 46 demonstrated in an LPS sepsis model that treatment with non‐senescent human BMSCs resulted in increased survival despite decreasing IL‐10 values. Although senescent human BMSCs were able to inhibit lymphocyte proliferation in vitro, the ability to positively influence sepsis was abrogated. In a recent study of CLP induced sepsis, Guldner et al. 69 reported a reduction in TNF‐α and in IL‐6 that was accompanied by a modest reduction in IL‐1β following injection of either 1 × 10^5^ human BMSCs (hBMSCs) or mouse BMSCs (mBMSCs). An increase in IL‐10 was observed in mice treated with hBMSC, whereas mBMSC treatment resulted in a decrease. Both groups displayed similar decreases in lung edema and inflammation; however, hBMSCs were superior in restoring lung function. Interestingly, the authors reported no difference on day 3 survival amongst untreated CLP animals (89%) and those treated with either hBMSC (82%) or mBMSC (96%). The role of MSC in modulating cytokine levels in sepsis syndrome models is undoubtedly complex; concerning IL‐10, conflicting results have shown MSCs to both positively 50, 56, 57, 63, 64, 67, 70, 73 as well as negatively 46, 54 influence levels, or alternatively have no effect 59, 60. There are similar inconsistencies in relation to IFN‐γ levels, with groups describing a reduction 50, 57, 73, an increase 70 or no effect 51, 67. It is likely that this apparent functional heterogeneity is a reflection of differences in experimental models, treatment regimens and MSC sources.

**Table 2:** Preclinical studies of mesenchymal stromal cells in treating ALI and sepsis in which mortality was assessed

Model	Treatment	Dose regimen	Result of cell treatment	Author, Year [Ref.]
Mouse, ALI, *E. coli*‐induced	Mouse BMSC (syngeneic)	7.5 × 10^5^ IT 4 hours post *E. coli* challenge	↑ survival, IL‐10 ↓ pulmonary edema, alveolar epithelial permeability ↓ TNF‐α and MIP‐2 Apoptotic MSC = no effect	Gupta et al. 2007 49
	Mouse BMSC apoptotic (syngeneic)			
Mouse, sepsis/colitis, CLP	Human ADSC	1 × 10^6^ IP 4 hours post CLP	↑ survival, IL‐10, bacterial clearance ↓ TNF‐α, IL‐6, IL‐1β, MIP‐2. RANTES, IL‐12, IFN‐γ	Gonzalez‐Rey et al. 2009 50
	Mouse ADSC (allo/syngeneic)			
Mouse, sepsis/colitis, LPS	Human ADSC	3 × 10^5^ or 1 × 10^6^ IP 0.5 hours post LPS		
Mouse, sepsis, CLP	Mouse BMSC (auto/allo)	1 × 10^6^ IV 24 hours prior or 1 hour post CLP	↑ survival, kidney function, IL‐10 ↓ TNF‐α, IL‐6 No change = IFN‐γ	Nemeth et al. 2009 51
Mouse, sepsis, CLP	Mouse BMSC (allo) +/− IL‐10 overexpression	1 × 10^6^ IV 1 hour post CLP	↑ survival ↓ TNF‐α, IL‐6, IL‐1α IL‐1β	Bi et al. 2010 [52]
Rat, ALI, bleomycin inhalation	Rat BMSC (allo)	1 × 10^6^ IV 96 hours post bleomycin	↑ survival ↓ IL‐1β, TGF‐β, VEGF,IL‐6, TNF‐α, NOS	Lee et al. 2010 [53]
Mouse, sepsis /ALI, CLP	Mouse BMSC (allo) +/− antibiotics	2.5 × 10^5^ IV 6 hours post CLP	↑ survival, bacterial clearance, organ function ↓ IL‐6, IL‐1β, IL10, KC, JE, CCL5	Mei et al. 2010 [54]
Mouse, ALI, *E. coli*‐induced	Human UC‐MSC	1 × 10^5^ IT 3 hours post *E. coli* challenge	↑ survival, bacterial clearance ↓IL‐1α, IL‐1β, IL‐6, TNF‐α, MIP‐1α, MIP‐1β, MIP‐2, RANTES, MPO	Kim et al. 2011 [55]
Rat, ALI, LPS	Rat BMSC (allo)	1 × 10^6^ IV 2 hours post LPS	↑ survival (not significant), IL‐10 ↓ IL‐1β, TNF‐α, MPO	Liang et al. 2011 [56]
Mouse, ALI, LPS	Human UC‐MSC	1 × 10^6^ IV 4 hours post LPS	↑ survival, IL‐10, Treg ↓ IFN‐γ, TNF‐α, MIP‐2	Sun et al. 2011 [57]
Rat, sepsis, CLP	Rat ADSC (auto)	1.2 × 10^6^ IV at 0.5, 6, & 18 hours post‐CLP	↑ survival, ↑ Tregs (healthy only) ↓ Tregs (apoptotic only) ↓ TNF‐α (apoptotic only)	Chang et al. 2012 58
	Rat ADSC apoptotic (auto)			
Mouse, ALI, *E. coli*‐induced	Mouse BMSC (allo)	7.5 × 10^5^ IT 4 hours post *E. coli* challenge	↑ survival, lipocalin 2 ↓ TNF‐α, MIP‐2, MPO No change = IL‐10	Gupta et al. 2012 [59]
Mouse, sepsis, *P. aeruginosa* induced	Human BMSC	1 × 10^6^ IV 1 hour post *P. aeruginosa* challenge	↑ survival, bacterial clearance ↓ PAI‐1 No change = TNF‐α, IL‐10, MIP‐2, PGE_2_	Krasnodembskaya et al. 2012 [60]
Rat, ALI, LPS	Human UC‐MSC	5 × 10^5^ IV 1 hour post LPS	↑ survival ↓ IL‐1β, TNF‐α, IL‐6, MPO No change = IL‐10	Li et al. 2012 [61]
Rat, VILI	Rat BMSC (allo)	4 × 10^6^ IV/IT 2.5–3 hours post VILI initiation	↑ lung recovery ↑ IL‐10 (IV BMSC only) ↑ KGF (IT BMSC only) ↓ TNF‐α, IL‐6 ↓ lung inflammation No change = survival	Curley et al. 2013 [62]
	Rat BMSC (allo)conditioned medium	300 μl IT 2.5–3 hours post VILI initiation		
Rat, ALI, Paraquat	Rat BMSC (allo) +/− methylprednisolone	1 × 10^7^ IV 6 hours post paraquat	↑ survival, IL‐10, SOD ↓ NF‐кB p65, TNF‐α, IL‐1β, IL‐6, MDA	Yang et al. 2013 [63]
Rat, ALI, LPS + chest impact	Rat BMSC (syngeneic)	2.5 × 10^6^ IV 2 hours post LPS	↑ survival, IL‐10 ↓ TNF‐α, IL‐6, No change = IL‐1β	Zhao et al. 2013 [64]
Mouse, sepsis, LPS	Human BMSC alive/senescent	1 × 10^6^ IV 0.5 hour post LPS	↑ survival ↓ TNF‐α, IL‐6, IL‐10senescent MSC = no effect	Sepúlveda et al. 2014 [46]
Rat, sepsis‐induced kidney injury, CLP	Rat ADSC apoptotic (auto) +/− melatonin	1.2 × 10^6^ IV at 0.5, 6, & 18 hours post‐CLP	↑ survival, kidney function ↓ TNF‐α, NF‐кB MIP‐1α, IL‐1β, RANTES No change = Treg	Chen et al. 2014 [65]
Rat, sepsis, CLP	Human BMSC	5 × 10^6^ IV 4 hours post CLP	↑ survival, Treg ↓ TNF‐α, IL‐6	Chao et al. 2014 [66]
	Human UC‐MSC			
Mouse, sepsis, *E. coli* induced	Mouse ADSC (auto)	1 × 10^6^ RO at time of *E. coli* challenge	↑ survival, IL‐10 ↓ TNF‐α, MPC‐1, IL‐6 No change = IL‐12, IFN‐γ	Pedrazza et al. 2014 [67]
Mouse, sepsis, CLP	Human MenSC +/− antibiotics	7.5 × 10^5^ IP 3 hours post CLP	↑ survival, bacterial clearance, liver function ↓ TNF‐α, MPC‐1, IL‐6, IL‐10	Alcayage‐Miranda et al. 2015 [68]
Mouse, sepsis/ALI, CLP	Human BMSC	1 × 10^5^ IV 24 hours post CLP	↑ lung recovery (human BMSC) ↑ IL‐10 (human BMSC) ↓ TNF‐α, IL‐6 ↓ IL‐10 (mouse BMSC) No change = survival	Guldner et al. 2015 [69]
	Mouse BMSC			
Rat, ALI, *E. coli* induced	Human BMSC	Series 1 1 × 10^7^ or 2 × 10 ^7^ IV 0.5 hours post *E. coli* challenge	↑ lung recovery, IL‐10, KGF ↑ bacterial clearance (2 × 10^7^ only) ↓ BAL neutrophils No change = IL‐6	Devaney et al. 2015 (43)
		Series 2 2 × 10^6^, 5 × 10^6^ or 1 × 10^7^ IV 0.5 hours post *E. coli* challenge	↑ survival, lung recovery, bacterial clearance ↑ IL‐10, KGF (1 × 10^7^ only) ↓ BAL neutrophils (1 × 10^7^ only) ↓ IL‐6	
		Series 3 1 × 10^7^ IV or IT 0.5 hours post *E. coli* challenge	↑ survival, lung recovery, bacterial clearance, IL‐10, KGF ↓ BAL neutrophils (IV only) ↓ IL‐6	
Mouse, sepsis, CLP	Mouse DMC (auto)	2 × 10^6^ IV 4 hours post CLP	↑ survival, IL‐4, IL‐5, IFN‐γ ↓ IL‐1β, IL‐6 No change = IL‐10	Wang et al. 2015 [70]
Rat, VILI	Rat BMSC (allo)	1 × 10^7^ IV 1.5 – 2.5 hours post VILI initiation	↑ lung recovery (IV BMSC only) ↓ lung inflammation (IV BMSC only) ↓ IL‐1β, IL‐6 (IV BMSC only) No change = survival, IL‐10, KGF	Hayes et al. 2015 [71]
	Rat BMSC (allo)conditioned medium	500 μl IV 1.5 – 2.5 hours post VILI initiation		
Mouse, ALI, *E. coli* induced	Human BMSC	8 × 10^5^ IT/IV 4 hours post *E. coli* challenge	↑ survival, bacterial clearance, KGF ↓ lung inflammation, protein permeability ↓ MIP‐2, TNF‐α	Monsel et al. 2015 [72]
	Human BMSC microvesicles	30, 60, or 90 μl IT/IV 4 hours post *E. coli* challenge		
Rat, sepsis‐induced organ injury, CLP	Human WJ‐MSC + antibiotics	1 × 10^6^ IP 6 hours post CLP	↑ survival, liver function, kidney function, IL‐4. IL‐10 ↓ NF‐кB, IL‐1α, IL‐6, IFN‐γ No change = TNF‐α	Cóndor et al. 2016 [73]
Rat, sepsis‐induced organ injury, cecal bacteria induced	Rat ADSC (auto) +/− antibiotics	5 × 10^5^ IV at 0.5, 6, & 18 hours post‐sepsis induction	↑ survival, kidney function, ↓ TNF‐1α, NF‐кB, IL‐1β, MMP‐9, RANTES ↓ ROS	Sung et al. 2016 74

Abbreviations: ADSC, adipose derived mesenchymal stem cell; ALI, acute lung injury; Auto, autologous; Allo, allogeneic; BMSC, bone marrow derived mesenchymal stem cell; CLP, cecal ligation and puncture; CM, conditioned medium; DMC, dermal‐derived mesenchymal cells; IP, intraperitoneal; IT, intratracheal; IV, intravenous; LPS, lipopolysaccharide; KGF, keratinocyte growth factor; MenSC, menstrual derived mesenchymal stem cell; RO, retro orbital; UC‐MSC, umbilical cord derived mesenchymal stem cell; VILI, ventilation‐induced lung injury; WJ‐MSC, Wharton's Jelly‐derived mesenchymal stem cell; ↑, an increase relative to baseline value; ↓, a decrease relative to baseline value.

## MSCs Promote Bacterial Clearance

Seemingly running contrary to their immunosuppressive capacity, treatment with MSCs has been reported to improve bacterial clearance 55, 60. Although MSC themselves lack phagocytic activity 54, MSCs can stimulate phagocytosis by monocytes 60, macrophages 54 and neutrophils 75. Gonzalez‐Rey et al. 50 reported that in addition to positively influencing the cytokine balance and animal survival, exposure to either human (1 × 10^5^−5 × 10^6^) or mouse (1 × 10^6^) ADSCs resulted in reduced peritoneal bacterial counts, possibly through modulating activated macrophage activity. MSCs also appear to inhibit bacterial growth through the secretion of antimicrobial compounds including the human cathelicidin antimicrobial peptide LL‐37 76, keratinocyte growth factor (KGF) 77, and lipocalin 2 59. Lee et al. 77 examined the effects of hBMSC in an ex‐vivo perfused human lung model. Application of hBMSC (5 or 10 × 10^6^ cells) either one or 2 hours after *E. coli* challenge (10^9^ or 10^10^ CFU) resulted in a significant decrease in bacterial load, improved alveolar fluid clearance and reduced inflammatory cell infiltration. MSCs antimicrobial effect was abolished following inhibition of KGF by neutralizing antibody. In a series of experiments performed by Devaney et al. 43 in a rodent *E. coli* ALI model, it shown that a single dose of between 5 × 10^6^ and 2 × 10^7^ of hBMSCs resulted in improved survival, reduced lung injury and a reduced bacterial burden that was also associated with increased LL‐37 concentrations. Intravenous and intratracheal administration routes were found to be equally effective in prolonging survival and alleviating ALI symptoms. However, hBMSC applied intravenously led to a reduction in the frequency of alveolar neutrophils while intratracheal hBMSC was more effective at increasing levels of IL‐10 and KGF.

## MSCs Enhance Lung Recovery

Multiple studies examining the effect of MSC in models of lung injury have reported MSC application to be associated with enhanced lung recovery and regain of function. Curley et al. 62 demonstrated in rat that following ventilation‐induced lung injury (VILI), intravenous or intratracheal administration of allogeneic BMSC (4 × 10^6^ cells) restored lung function, enhanced the alveolar air‐space volume and reduced alveolar thickening and decreased markers of inflammation. A similar effect was also reported following intratracheal administration of MSC conditioned media (CM). However, in a follow‐up study performed by the same group, intravenously administered CM was found to be largely ineffective at restoring arterial oxygenation, respiratory static compliance, lung wet:dry ratio and reducing inflammation when compared to intravenously administered allogeneic BMSC (1 × 10^7^ cells) 71.

## Recent MSC‐Derived Therapeutic Strategies

Cellular‐based therapies currently in development for sepsis syndrome and ALI/ARDS treatment have focused on MSC derived from bone marrow or adipose tissue. However, recent studies have also assessed the feasibility of utilizing MSC isolated from alternative sources whilst others are examining the therapeutic efficacy of MSC‐derived vesicles. In a recent publication, Cóndor et al. 73 analyzed the efficacy of MSCs derived from human Wharton's Jelly (WJ‐MSCs) in a rat sepsis model. In animals receiving 1 × 10^6^ WJ‐MSC 6 hours after CLP, 5‐day survival was significantly increased (87.5% vs. 55.6% in CLP only) and both liver and kidney function were improved. WJ‐MSCs treatment resulted in IL‐1α, IL‐6, and IFN‐γ values similar to non‐CLP controls. However, there was no detectable effect on either IL‐4 or IL‐10. In a CLP mouse sepsis model, Alcayaga‐Miranda et al. 68 evaluated human MSCs derived from menstrual fluid (MenSC) in combination with antibiotic therapy. Injection of 7.5 × 10^5^ MenSC cells promoted survival and bacterial clearance, improved lung histology and was associated with a decrease in markers of multiorgan dysfunction. A synergistic effect was observed when MenSC were combined with Enrofloxacin. At 40 hours, cell‐treated animals exhibited reduced TNF‐α, IL‐6, MCP1, and also IL‐10. The authors reported that while LL‐37 could not be detected, expression of the peptide hormone hepcidin was increased in treated animals, inhibition of which nullified the antimicrobial effect. Wang et al. 70 reported on the ability of murine, nonexpanded, dermal‐derived MSCs (DMCs) to attenuate CLP–induced sepsis. Up to 1 × 7^10^ DMCs were recovered from processed dermis samples without the need for further culture. Mice injected with 1 × 10^6^ DMCs 4 hours after CLP possessed decreased IL‐1β and IL‐6 values and displayed an increase in IL‐4, IL‐5, and IFN‐γ. However, no significant change in IL‐10 level was detected. In addition, DMCs positively influenced macrophage migration and phagocytic activity. At present, preclinical and clinical studies of MSC‐based technologies have focused on the use of whole MSCs. However, there is growing body of evidence that extracellular vesicles (EVs), a constitute of MSC CM that includes endosomes and microvesicles, might impart a therapeutic benefit through transporting and delivering MSC‐derived protein, microRNA and mitochondria to target cells. The therapeutic capacity of hBMSC‐derived MVs was recently compared to that of MSC by Monsel and colleagues 72. MVs were recovered from a 48‐hour culture of 9 × 10^6^ hBMSC by ultracentrifugation. Similar beneficial effects to *E. coli* induced ALI were observed in response to MV and MSC intervention. Survival and bacterial clearance was increased in both groups and lung injury was decreased. Preincubation of hBMSC MVs with anti‐CD44 antibody impaired the effect on survival, suggesting that cellular uptake of MVs is required in order to elicit a therapeutic effect. Although a detailed assessment of MV‐based therapies is beyond the scope of this current review, Monsel et al. 78 has produced a particular thorough examination of the scientific literature in this area.

## MSC in Acute Respiratory Distress and Sepsis Syndromes—The Clinical Experience

Currently, there are only a limited number of reports detailing the response of septic and ALI/ARDS patients to MSC. However, there are a number of ongoing clinical trials registered which are estimated to be completed shortly or within the next 1–2 years (Table 3). One of the earliest studies to examine the safety of MSCs in ARDS patients was conducted by Zheng et al. (NCT01902082) 79. In this phase I, single‐center, double‐blind, placebo‐controlled study, 12 patients were randomized 1:1 and received a peripheral intravenous infusion of either 100 ml saline or a single dose of allogeneic ADSC equivalent to 1 × 10^6^ cells kg/bw over 1 hour. The study reported that while ADSC infusion appeared safe, there were no significant differences in total length of hospital stay, ICU‐free days, and ventilator‐free days or in serum ARDS biomarkers (SP‐D, IL‐6, IL‐8) between treatment arms. However, the authors acknowledged that the study's small sample size and a limited follow‐up period of 28 days hampered further analyses. The RUMCESS (NCT01849237) trial, a single‐center, open label, randomized study, sought to assess the impact of MSCs in patients with septic shock and severe neutropenia. The primary outcome measure was 28‐day mortality; secondary outcome measures included the effect of MSC on organ dysfunction parameters, systemic inflammatory markers and SOFA‐score. Of the 27 patients enrolled, 13 received conventional treatment while 14 received conventional treatment plus a single dose of 1 × 10^6^ MSC IV administered within 10 hours after onset. Galstian et al. 80 reported that the MSC treated group had a significant increase in 28‐day survival rates (57% vs. 15%) that was associated with a decrease in SOFA‐score. However, there was no difference in post‐28 day survival rates. While an improvement in 28‐day survival is certainly encouraging, it is difficult to draw further conclusions from this study as no inflammatory marker analysis was performed and there is little information on the type of MSCs used in the study. CELLULA (NCT02328612) was a commercially sponsored, randomized interventional trial designed to study the effects of ADSC in healthy male volunteers treated with LPS. After receiving LPS, 32 volunteers received either IV placebo or 2.5 × 10^5^, 1 × 10^6^ or 4 × 10^6^ cells kg/bw of Cx611, an allogeneic ADSC. Though the trial was registered as complete in April 2015, at the time of writing no peer reviewed results are available. In 2015, Wilson et al. 81 published results of a phase I, multicenter, open label, dose‐escalation pilot study, START (NCT01775774). Selecting patients with moderate to severe ARDS, the aim of this study was to assess the safety of allogeneic bone‐marrow derived MSCs (BMSC) by measuring the incidence of defined prespecified infusion associated events experienced by three cohorts (9 patients in total) receiving either 1 × 10^6^, 5 × 10^6^, and 10 × 10^6^ cells kg/bw. The study concluded that while all BMSC dose levels were well tolerated, no significant differences in ARDS markers (IL‐6, IL‐8, ANGPT2, and AGER) between cohorts could be detected. A follow‐up phase II efficacy trial is currently ongoing (NCT02097641). Simonson et al. 82 reported on the clinical outcome of two patients diagnosed with severe ARDS treated with 2 × 10^6^ cells kg/bw allogeneic BMSC. Cell infusion in both patients was uneventful and was completed without complication. At 5 days post‐infusion, patient 1 developed nosocomial pneumonia that subsequently responded to antibiotic treatment. Patient 1 was extubated 4 weeks after MSC infusion and patient 2 after 12 days. Both patients showed a decrease in markers of epithelial apoptosis, alveolar‐capillary fluid leakage together with a decline in pro‐inflammatory cytokines, miRNAs, and chemokines in plasma and BAL fluid. The authors acknowledged that the treatment of these patients generated valuable data, but that larger patient cohorts would be required to thoroughly investigate the response of ARDS patients to MSCs.

**Table 3:** Ongoing and new studies of MSC in sepsis syndrome and ALI/ARD

Study title	Indication	Registry numbers	Study design	Estimated enrolment (to received cell therapy)	Cell type	Dosing schedule	Start Date	Est. End Date
A pilot study for the efficacy and safety of mesenchymal stem cell in acute severe respiratory failure (STELLAR‐Pilot).	ARDS	NCT02112500	Phase II, open label, single group assignment	10 (10)	Auto BMSC	Unknown	Feb 2014	Dec 2016
Treatment of severe acute respiratory distress syndrome with allogeneic bone marrow‐derived mesenchymal stromal cells (MSC‐ARDS).	ARDS	NCT02215811	Phase I, open label, single group assignment	10(10)	Allo BMSC	Unknown	Mar 2014	Dec 2015
Phase1 study of recombinant stem cells that repair lung injury in H7N9 infected patients.	ALI/ARDS	NCT02095444	Phase I/II, open label, single group assignment	20(20)	MenSC	Four infusion; 1 × 10^7^ kg/bw IV (twice per week)	Mar 2014	Dec 2016
Prospective, randomized, multicenter phase 2 clinical trial of allogeneic bone marrow‐derived human mesenchymal stem cells for the treatment of acute respiratory distress syndrome (START).	ARDS	NCT02097641	Phase II, randomized, double blind, single group assignment	60 (30)	Allo BMSC	Single infusion; 1 × 10^6^ kg/bw IV day one	Mar 2014	Dec 2017
Cellular immunotherapy for septic shock: A phase I trial (CISS).	Sepsis	NCT02421484	Phase I, open label, single group assignment	9 (9)	Allogeneic BMSC	Single infusion, IV Cohort 1, 3 × 10^5^ kg/bw Cohort 2, 1 × 10^6^ kg/bw Cohort3, 3 × 10^6^ kg/bw	May 2015	Sep 2016
Safety and efficacy of human umbilical‐cord‐derived mesenchymal stem cell transplantation in acute lung injury (UCMSC‐ALI).	ALI	NCT02444455	Phase I/II, open label, single group assignment	20 (20)	Allogeneic UCSC	Three infusions; 5 × 10^5^ kg/bw IV	May 2015	Dec 2017
A phase Ib/IIa, randomized, double blind, parallel group, placebo controlled, multicenter study to assess the safety and efficacy of expanded Cx611 allogeneic adipose‐derived stem cells (eASCs) for the intravenous treatment of adult patients with severe community‐acquired bacterial pneumonia and admitted to the intensive care unit (SEPCELL).	Sepsis	2015‐002994‐39	Phase I/II, randomized, double blind, parallel assignment	180 (90)	Allogeneic ADSC	Two infusions; 1.6 × 10^7^ cells day 1 1.6 × 10^7^ cells day 3	Oct 2015	Jul 2017
A phase 1/2 study to assess the safety and efficacy of multistem therapy in subjects with acute respiratory distress syndrome (MUST‐ARDS).	ARDS	NCT02611609	Phase I/II, double blind, parallel assignment	36 (26)	Allogeneic MAPC	Cohort 1, 3 × 10^6^ cell Cohort 2, 9 × 10^6^ cell Cohort 3, either 3 × 10^6^ or 9 × 10^6^ cell	Jan 2016	Dec 2017
A phase II trial of mesenchymal stem cells for treatment of acute respiratory distress syndrome in stem cell transplant patients.	ARDS	NCT02804945	Phase II, randomized, double blind, parallel assignment	50 (25)	Allogeneic MSC (source unknown)	Single infusion; 1 × 10^7^ kg/bw IV day one	Oct 2016	Oct 2018

Abbreviations: ADSC, adipose derived mesenchymal stem cell; ALI, acute lung injury; Allo, allogeneic; ARDS, acute respiratory disease syndrome; Auto, autologous; BMSC, bone marrow derived mesenchymal stem cell; IV, intravenous; MenSC, menstrual derived mesenchymal stem cell; UCDS, umbilical cord derived mesenchymal stem cell.

## Conclusion

Sepsis syndrome and ALI/ARDS represent a significant treatment challenge and remain a frequent course of death. Despite extensive efforts to develop innovative therapeutic strategies, none so far have translated into a new evidence‐based treatment. Compounds which repeatedly showed promise in preclinical and phase I/II trials, have subsequently failed phase III assessment. Most notably human recombinant APC, the only compound to receive marketing authorization for sepsis treatment, was subsequently withdrawn after follow‐up phase III studies failed to replicate the patient benefit observed in its initial registration trial 24, 83. It is hardly surprising then, that research into anti‐inflammatory therapeutics for sepsis treatment has been referred to as a “graveyard for pharmaceutical companies” 84. Yet studies into MSC‐based therapies in sepsis and ALI/ARDS are encouraging and the majority of preclinical results confirm that MSCs are capable of dampening the early pro‐inflammatory cascade, decreasing infection and improving survival. Notwithstanding, when interpreting these results, particular attention should be paid to the animal model used. Is the model sufficient and capable of accurately reflecting the clinical complexities of the disease course? Certainly, a criticism that can be leveled at the majority of preclinical research into these syndromes is a heavy reliance on results obtained from small inbred animal models which are radically dissimilar to the diverse septic patient demographic. Large animal models including baboon, pig, and sheep have been developed for the study of ALI/ARDS and these may more accurately represent various clinical facets of the human disease course. Indeed, encouraging results have been reported in porcine 85 and ovine 86 ALI/ARDS models regarding the effects of MSC treatment. However as preclinical research programs utilizing large animal models might take longer to complete, be more costly and require further specialized handling requirements, there usage is somewhat limited. Animal models utilize an artificially induced sepsis that, in order to aid further experimental analysis, is typically accelerated, clearly defined, and reproducible. Finally, in models designed to assess the impact MSCs, cell therapy is typically applied before or at the time of sepsis induction; a treatment regimen which is clearly unrealistic for the majority of septic patients. The results from early clinical trials have provided tantalizing hints that MSCs can provide a therapeutic benefit. However, further interpretation of the effect of MSC in sepsis syndrome is hindered due to small sample sizes, difference in experimental design and biomarker selection. We contend that future trials consider including standardized immunological analysis of treated patients to simplify inter‐study comparisons and promote pooling of data. For example, DuraClone IM panels 87 are increasingly being used within studies of cellular therapy in solid organ transplantation 47, 88. The next generation of MSC sepsis studies will need to take into account the innate heterogeneity of sepsis syndrome and clearly patient selection and stratification will be of paramount importance. To address this issue within our own planned phase I/II study of third‐party MSC‐based product in sepsis syndrome, we will select only those patients presenting with sepsis of an abdominal origin that occurs following surgical interventional and/or source control. While there is currently insufficient clinical evidence concerning the efficacy of MSC in the treatment of sepsis syndrome and ARDS, results from recently completed clinical trials are expected shortly and may clarify what the future holds for MSC‐based therapeutics.

## Author Contributions

C.L.J., Y.S., and M.H.D.: manuscript writing, final approval of manuscript.

## Disclosure of Potential Conflicts of Interest

The authors indicate no potential conflicts of interest.
